# Pathogenic D76N Variant of β_2_-Microglobulin: Synergy of Diverse Effects in Both the Native and Amyloid States

**DOI:** 10.3390/biology10111197

**Published:** 2021-11-17

**Authors:** Éva Bulyáki, Judit Kun, Tamás Molnár, Alexandra Papp, András Micsonai, Henrietta Vadászi, Borbála Márialigeti, Attila István Kovács, Gabriella Gellén, Keiichi Yamaguchi, Yuxi Lin, Masatomo So, Mihály Józsi, Gitta Schlosser, Young-Ho Lee, Károly Liliom, Yuji Goto, József Kardos

**Affiliations:** 1 ELTE NAP Neuroimmunology Research Group, Department of Biochemistry, Institute of Biology, ELTE Eötvös Loránd University, 1117 Budapest, Hungary; evi.bulyaki@gmail.com (É.B.); juditkun.bc@gmail.com (J.K.); micsonai@ttk.elte.hu (A.M.); vadaszih@gmail.com (H.V.); 2 Department of Biochemistry, Institute of Biology, ELTE Eötvös Loránd University, 1117 Budapest, Hungary; molnar.tamas@ttk.elte.hu (T.M.); marialigeti.borbala@gmail.com (B.M.); koviatis94@gmail.com (A.I.K.); 3 Complement Research Group, Department of Immunology, ELTE Eötvös Loránd University, 1117 Budapest, Hungary; papp.alexandra93@freemail.hu (A.P.); mihaly.jozsi@ttk.elte.hu (M.J.); 4 Department of Analytical Chemistry, Institute of Chemistry, ELTE Eötvös Loránd University, 1117 Budapest, Hungary; gabgellen@staff.elte.hu (G.G.); gitta.schlosser@ttk.elte.hu (G.S.); 5 Global Center for Medical Engineering and Informatics, Osaka University, Osaka 565-0871, Japan; kyamaguchi@mei.osaka-u.ac.jp (K.Y.); gtyj8126@protein.osaka-u.ac.jp (Y.G.); 6 Research Center of Bioconvergence Analysis, Korea Basic Science Institute (KBSI), Ochang 28119, Korea; linyuxi@kbsi.re.kr (Y.L.); mr0505@kbsi.re.kr (Y.-H.L.); 7 Institute for Protein Research, Osaka University, Osaka 565-0871, Japan; mso@protein.osaka-u.ac.jp or; 8 MTA-ELTE Complement Research Group, Eötvös Loránd Research Network (ELKH), Department of Immunology, ELTE Eötvös Loránd University, 1117 Budapest, Hungary; 9 Bio-Analytical Science, University of Science and Technology (UST), Daejeon 34113, Korea; 10 Graduate School of Analytical Science and Technology (GRAST), Chungnam National University (CNU), Daejeon 34134, Korea; 11 Research Headquarters, Korea Brain Research Institute (KBRI), Daegu 41068, Korea; 12 Department of Biophysics and Radiation Biology, Faculty of Medicine, Semmelweis University, 1094 Budapest, Hungary; liliom.karoly@med.semmelweis-univ.hu

**Keywords:** amyloidosis, protein aggregation, β_2_-microglobulin, dialysis-related amyloidosis, protein stability, ion-pairs, differential scanning calorimetry, CD spectroscopy

## Abstract

**Simple Summary:**

Elevated β_2_-microglobulin (β2m) serum levels cause serious complications in patients on long-term kidney dialysis by depositing in the form of amyloid fibrils in the osteoarticular system. Recently, a hereditary systemic amyloidosis was discovered, caused by a naturally occurring D76N β2m mutant exhibiting normal serum levels and a distinct, visceral deposition pattern. D76N β2m showed a structure remarkably similar to the wild-type (WT) protein, albeit with decreased thermodynamic stability and increased amyloidogenicity. Despite the extensive research, the molecular bases of the aberrant aggregation of β2m in vivo remains elusive. Here, using a variety of biophysical techniques, we investigated the role of the pathogenic D76N mutation in the amyloid formation of β2m by point mutations affecting the stabilizing ion-pairs of β2m. We found that, relative to WT β2m, the exceptional amyloidogenicity of the pathogenic D76N β2m variant is realized by the synergy of diverse effects of destabilized native structure, higher sensitivity to negatively charged amphiphilic molecules and polyphosphate, more effective fibril nucleation, higher conformational stability of fibrils, and elevated affinity for extracellular matrix proteins. Understanding the underlying molecular mechanisms might help to find target points for effective treatments against diseases associated with the deleterious aggregation of proteins.

**Abstract:**

β_2_-microglobulin (β2m), the light chain of the MHC-I complex, is associated with dialysis-related amyloidosis (DRA). Recently, a hereditary systemic amyloidosis was discovered, caused by a naturally occurring D76N β2m variant, which showed a structure remarkably similar to the wild-type (WT) protein, albeit with decreased thermodynamic stability and increased amyloidogenicity. Here, we investigated the role of the D76N mutation in the amyloid formation of β2m by point mutations affecting the Asp76-Lys41 ion-pair of WT β2m and the charge cluster on Asp38. Using a variety of biophysical techniques, we investigated the conformational stability and partial unfolding of the native state of the variants, as well as their amyloidogenic propensity and the stability of amyloid fibrils under various conditions. Furthermore, we studied the intermolecular interactions of WT and mutant proteins with various binding partners that might have in vivo relevance. We found that, relative to WT β2m, the exceptional amyloidogenicity of the pathogenic D76N β2m variant is realized by the deleterious synergy of diverse effects of destabilized native structure, higher sensitivity to negatively charged amphiphilic molecules (e.g., lipids) and polyphosphate, more effective fibril nucleation, higher conformational stability of fibrils, and elevated affinity for extracellular components, including extracellular matrix proteins.

## 1. Introduction

Protein misfolding events often lead to the formation of protein aggregates, including highly ordered amyloid fibrils [1,2]. Amyloid fibrils can deposit as amyloid plaques in various tissues throughout the body, resulting in a wide range of pathological conditions [3]. Point mutations of amyloidogenic proteins (Aβ [4], transthyretin [5], apolipoprotein [6], gelsolin [7], and synuclein [8]) are known to be responsible for several familial amyloid disorders. These mutations can provoke protein aggregation by changing the protein’s physico-chemical properties or the stability of the native structure via changes in charge network, hydrophobicity, and propensity to form an intermolecular β-sheet structure [9,10,11,12,13,14,15,16].

β_2_-microglobulin (β2m), the light chain of the MHC-I complex, exhibiting a typical immunoglobulin fold, is involved in various human diseases (hemochromatosis, lymphoma, kidney diseases, etc.) [17,18]. Three decades ago, dialysis-related amyloidosis (DRA) was discovered among hemodialysis patients, which was linked to the deposition of wild-type (WT) β2m in amyloid plaques [19]. In 2012, a hereditary systemic amyloidosis of β2m was reported to be associated with the naturally occurring D76N β2m variant [20], which showed a structure remarkably similar to the WT protein, albeit with decreased thermodynamic stability and increased amyloidogenicity [21,22]. Remarkable differences were found between the pathology of the two β2m amyloidoses. The amyloid formation of the WT protein in DRA is associated with dramatically increased β2m serum levels and mainly involves the osteoarticular tissues, whereas the extensive amyloid deposits of D76N β2m are present in internal organs of mutation carriers. The aggregation of the mutant form is not related to increased β2m serum levels and reduced renal functions [20].

Recently, several studies have investigated the structure and amyloid forming propensity of the D76N β2m variant [23,24,25,26,27], but it is still not clear how this single point mutation may affect the amyloidogenic properties and cause a disease different from that caused by the WT protein. A recent investigation of β2m fibrils by ssNMR suggests rearrangement of intramolecular interactions in D76N β2m, which might be responsible for the elevated amyloidogenic propensity of the protein [28]. The cryo-EM structure of the WT β2m fibrils (grown at pH 2.5) shows residue 76 in the L-shaped subunit core, in a region stabilized by hydrophobic interactions [29]. At the same time, some promising strategies have emerged to inhibit the amyloid formation of D76N with pentraxins [30] or a single domain antibody [31].

In the case of DRA, elevated serum concentration of WT β2m is essential for protein aggregation, but data from several studies suggest that other macromolecules such as heparin [32,33], collagen [34], or lysophospholipids [35] might also play a crucial role in the process. Previously, we demonstrated that an inflammation-related signal molecule, lysophosphatidic acid (LPA), which circulates in high concentrations in the serum of DRA patients, specifically promotes amyloid formation of β2m and stabilizes amyloid fibrils [36]. Although D76N β2m was proven to be able to aggregate without additives under physiological conditions in vitro [20], biomolecules potentially interacting with the protein can have a significant impact on the mutant form’s aggregation.

In the present work, we investigated charge clusters on the surface of β2m, which are essential for stabilizing the native structure; their mutation might alter the amyloidogenicity and interaction network of β2m. We introduced point mutations affecting these clusters, especially focusing on the role of the disease-related 76Asp-41Lys ion-pair. We investigated the structure and conformational stability of the variants in both their native and amyloid form. Furthermore, we investigated the intermolecular interactions of the WT and mutant proteins with several potential binding partners, such as LPA, SDS, polyphosphate, collagen, and extracellular matrix proteins. Our results reveal that the distinct characteristics of the hereditary β2m amyloidosis can be explained by the synergy of diverse effects: decreased stability of the mutant monomer, increased sensitivity to destabilizing additives, higher stability of the amyloid fibrils, and importantly, an altered intermolecular interaction network.

## 2. Materials and Methods

### 2.1. DNA Constructs, Protein Expression, and Purification

Mutants of recombinant β2m were created with the method of megaprimer mutagenesis. All constructs were verified by nucleotide sequencing. For protein expression, we used E. coli Bl21 (DE3) strain with IPTG induction. The renaturation process of recombinant β2m variants was carried out as previously described [37]. After renaturation, we purified the variants by anion exchange chromatography (HiTrap Q HP column, GE Healthcare) in 10 mM Tris, 10 mM NaCl pH 7.4 buffer and eluted them using a NaCl gradient (10–500 mM). The purity of the proteins was verified by SDS-PAGE. The molecular mass of the β2m variants was confirmed by ESI-MS/MS. The protein concentration was determined by using the extinction coefficient of 20,065 M^−1^cm^−1^ at 280 nm.

### 2.2. Differential Scanning Calorimetry (DSC)

DSC measurements were carried out on a VP-DSC instrument (Microcal, Northampton, MA, USA). The protein concentration was 0.2 mg/mL in 50 mM Na-phosphate, 100 mM NaCl pH 7.4 buffer. Denaturation curves were recorded between 10–90 °C, with a scan rate of 1 °C/min, and the reversibility of the denaturation was demonstrated by repeated heating. Unfolding enthalpy changes were calculated by fitting to the baseline-corrected, normalized curves, assuming a two-state reversible system, using the built-in function of MicroCal Origin 7.0 software (Microcal, Northampton, MA, USA).

### 2.3. Circular Dichroism (CD) Spectroscopy 

CD spectra of β2m variants (0.1 mg/mL in 10 mM phosphate, 10 mM NaCl pH 7.4) were collected with a Jasco J-810 spectropolarimeter (Jasco Inc., Tokyo, Japan) equipped with a Peltier temperature controller. In the range of 190–250 nm, three scans were accumulated, with a scan rate of 10 nm/min, response time of 4 s, and bandwidth of 1 nm in a 1 mm pathlength cell at 37 °C. To study the effect of LPA and SDS on the native structure, 20–30 CD spectra of β2m samples (0.1 mg/mL in 50 mM Na-phosphate, 100 mM NaCl pH 7.4) containing various concentrations of LPA and SDS (250–500 μM) were collected at 6 min intervals at 37 °C.

### 2.4. Thioflavin T Fluorescence Assay

Amyloid fibril formation of 0.1 mg/mL β2m variants in the presence of 300 µM LPA, 250 or 500 µM SDS, or concentration series of 3–300 μM LPA or 25–250 μM SDS in 50 mM Na-phosphate, 100 mM NaCl, pH 7.4, was studied at 37 °C. In seeded experiments, the reactions were induced by the addition of 5 μg/mL ultrasonicated amyloid fibrils. The growth of amyloid fibrils was followed by monitoring the fluorescence of 10 μM ThT using 445 nm and 485 nm excitation and emission wavelengths, respectively, for 20–24 h in a Synergy H4 plate reader (BioTek Instruments Inc., Winooski, VT, USA) or on a Fluoromax Spex fluorimeter (Metuchen, NJ, USA).

Amyloid formation of β2m variants in the presence of polyphosphate (poly-P, consisting of 60 to 70 phosphate groups) under neutral conditions (pH 7.4) at 37 °C was monitored with a Spectramax iD3 Microplate reader (Molecular Devices LLC., San Jose, CA, USA. Stock solutions of β2m samples in 10 mM Na-phosphate, 10 mM NaCl, pH 7.4, were ultracentrifuged prior to the measurements (105,000× *g*, 30 min at 4 °C). Composition of the reaction mixtures were 0.3 mg/mL β2m, 20mM Tris (pH 7.4), 0.1 mM poly-P, and 2 μM ThT. Data were collected every 10 min. The samples were shaken (517 rpm) between the measurements, and no seeds were used.

### 2.5. Equilibrium Monomer Concentrations—Intrinsic Trp Fluorescence

Monomeric β2m stock solutions were ultracentrifuged at 105,000× *g* for 30 min at 4 °C to remove any preexisting protein aggregates. Triplicates of seeded protein samples (0.1 mg/mL overall protein concentration, in 50 mM Na-phosphate, 100 mM NaCl, pH 7.5, including 5 μg/mL ultrasonicated preformed fibrils) were incubated for 7 days at 37 °C under agitation (200 rpm) in the presence of 25–250 μM SDS or 3–300 μM LPA. Then, amyloid fibrils and aggregates were removed by ultracentrifugation at 105,000× *g* for 60 min. Supernatants were collected and analyzed by measuring intrinsic Trp fluorescence on a Fluoromax Spex fluorimeter (Metuchen, NJ, USA) immediately. Fluorescence emission spectra were recorded with excitation at 295 nm in the range of 310–400 nm using a 3 × 3 mm fluorescence cell. Fluorescence measurements were carried out at 25 °C.

Area under the curve was calculated for each spectrum using Origin 8.5 software (Microcal, Northampton, MA, USA). Calibration curves were prepared for standard β2m monomeric solutions (0.20–100 μg/mL) by linear fitting, and the equilibrium monomer concentration was calculated for each amyloid sample. Conformational stability (Δ*G*_a_) of amyloid fibrils was calculated from the equilibrium monomer concentrations, as described in Section 2.7.1.

### 2.6. Conformational Stability of the Native State of β2m Studied by GdnHCl Denaturation

β2m monomers in 50 mM Na-phosphate, 100 mM NaCl, pH 7.4 buffer were incubated for 12 h at 37 °C in the presence of increasing concentrations of GdnHCl (0–6 M). Then, CD spectra were recorded between 200–250 nm, and the intensities at 212 nm were used for analysis. To quantify conformational stability parameters, curves were analyzed following the methodology of Pace and Scholtz [38], using the following formula for fitting in Origin 8.5 software:

*y* = (*A*_N_ + *A*_D_ exp[–(Δ*G*° + *m* · *x*)/(*R* · *T*)])/(1 + exp[–(Δ*G*° + *m* · *x*)/(*R* · *T*)]) (1)

where *A*_N_ and *A*_D_ are the ellipticities of the native and denaturated molecules, Δ*G*° is the Gibbs free energy change of unfolding in the absence of GdnHCl, *m* is the slope of Δ*G* in the function of GdnHCl concentration, assuming a linear relationship, *R* is the gas constant, and *T* is the temperature.

### 2.7. Conformational Stability of β2m Amyloid Fibrils

#### 2.7.1. Calculation of Amyloid Fibril Stability from the Equilibrium Monomer Concentration

For simplicity, we applied the linear polymerization model [39,40,41,42], supposing that the equilibrium constant for adding a monomer to the end of a chain, *K*_a_, is independent of length:

$$\begin{array}{c} M+M\overset{K_{a}}{⇄}P_{2}\\ P_{2}+M\overset{K_{a}}{⇄}P_{3}\\ P_{i-1}+M\overset{K_{a}}{⇄}P_{i} \end{array}$$

where *M* is the monomer, and *P_i_* is polymer with size *i*. The association constant, *K*_a_ is defined by

(2) $$K_{a}=\frac{\left[P_{i}\right]}{\left[P_{i-1}\right]\left[M\right]}$$

Thus, the total concentration of β2m, [*M*]_0_, is expressed by

(3) $${\left[M\right]}_{0}=\overset{\infty }{\underset{i=1}{\sum }}i\left[P_{i}\right]=\overset{\infty }{\underset{i=1}{\sum }}iK_{a}^{i-1}{\left[M\right]}^{i}=\frac{\left[M\right]}{{\left(1-K_{a}\left[M\right]\right)}^{2}}$$

The advantage of this simplified model is that we can determine *K*_a_ value, which provides us the standard Gibbs free energy of association (Δ*G*_a_).

(4) $$K_{a}=\frac{1-\sqrt{\left[M\right]/{\left[M\right]}_{0}}}{\left[M\right]}$$

Δ*G*_a_ = −*R T* ln *K*_a_ (5)

#### 2.7.2. Stability of Amyloid Fibrils against GdnHCl Denaturation

To study the stability of amyloid fibrils against chemical denaturants, we monitored the ThT fluorescence intensity decrease of β2m aggregates in the presence of 0–7 M GdnHCl using a Synergy H4 plate reader (BioTek Instruments Inc., Winooski, VT, USA). Triplicates of amyloid fibrils grown in 50 mM Na-phosphate, 100 mM NaCl, pH 7.4 buffer in the presence of 300 µM LPA or 250 µM SDS were denatured following the procedures above. Samples were diluted tenfold in 10 µM ThT, 50 mM glycine, 100 mM NaCl, pH 8.5 buffer and measured in duplicate on 96-well fluorescence plates (Greiner Bio-One, Frickenhausen, Germany). The monomer protein fraction was determined from the decrease in ThT intensities relative to the maximum values. *K*_a_ association constants and Δ*G*_a_ values as a function of the GdnHCl concentration were calculated by using the linear polymerization model, as described in Section 2.7.1. Assuming a linear dependence on the GdnHCl concentration, Gibbs free energy of association in the absence of denaturant (Δ*G*_a_^0^) was calculated from

Δ*G*_a_ = Δ*G*_a_^0^ + *m* [GdnHCl] (6)

### 2.8. ELISA Assay for β2m Binding to Extracellular Matrix Proteins

Microtiter 96-well microplates (Thermo Scientific) were coated for 1 h with 10 µg/mL Maxgel (Sigma-Aldrich), collagen I from human placenta (Sigma-Aldrich), osteoadherin, and fibromodulin in DPBS buffer. Osteoadherin and fibromodulin were kind gifts from Anna M. Blom, Department of Translational Medicine, Lund University, Malmö, Sweden. Wells were washed and blocked with 5% (*w*/*v*) bovine serum albumin (BSA, Sigma-Aldrich) in DPBS buffer with 0.05% Tween 20 for 1 h. After washing three times, the β2m variants were added to the wells in serial dilution with the range of 0–10 µg and incubated for 1 h. The detection was carried out using mouse anti-human β2m (Biolegend, 316302) and goat anti-mouse-HRP conjugated (Dako, P0447) antibodies. Absorbance was detected at 450 nm.

## 3. Results and Discussion

### 3.1. The Role of the Ion-Pairs in the Stability of Native β2m

**Figure 1:**
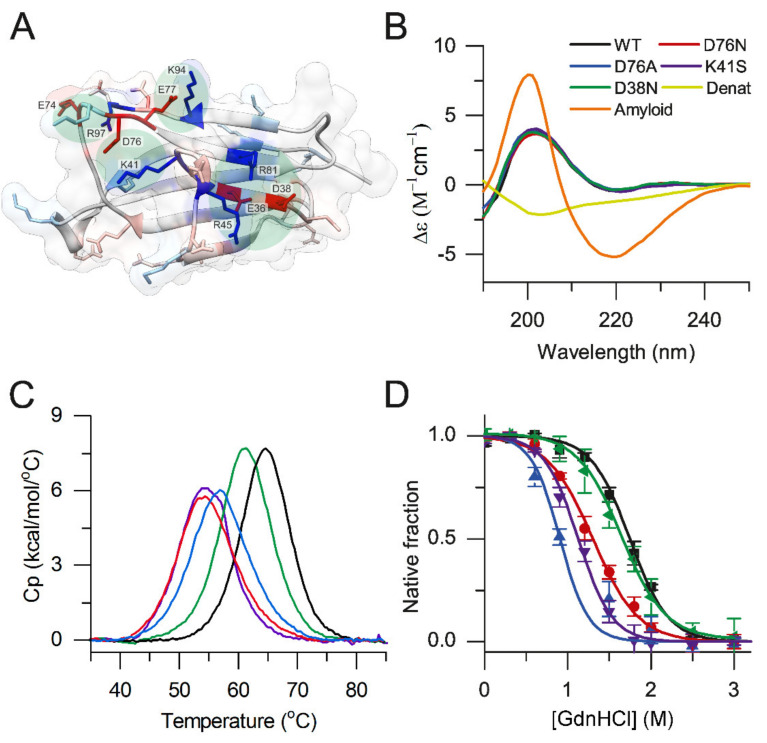
(**A**) Charged residues at the surface of β2m. Asp and Glu residues are shown in red, while Lys and Arg are shown in blue. Ion-pairs and clusters consisting of sequentially distant residues are highlighted and labeled. In the present work, we focused on the role of the Asp76-Lys41 ion-pair and charge cluster around Asp38. (**B**) CD spectra of native β2m variants were measured in 10 mM Na-phosphate, 10 mM NaCl, pH 7.4. For comparison, the acid denatured and amyloid fibril spectra of WT β2m were recorded at pH 2.5 in 50 mM citrate buffer. (**C**) Thermal denaturation of β2m variants were measured by DSC in 50 mM Na-phosphate, 100 mM NaCl, pH 7.4. (**D**) Chemical denaturation of the variants in the same buffer was followed by CD spectroscopy in the presence of 0–6 M GdnHCl at 37 °C. Mean ± S.E.M. Color codes in (**C**,**D**) are the same as in (**B**).

Clusters of charged side-chains are known to affect protein stability, amiloidogenic profile, and interaction network [43]. Twenty-eight charged side-chains at the surface of β2m might contribute to the high solubility of the protein and may have a role in electrostatic interactions with partner molecules. However, the number of charged residues forming intramolecular ion-pairs or charge clusters is low (Supplementary Table S1). We identified the ion-pairs that are formed by sequentially distant side chains. We believe these might play a role in the overall fold, stability, and dynamics of the β2m molecule via electrostatic linkages. There are only four such clusters or ion-pairs, the Lys41-Asp76 salt bridge, the charge cluster centered around Asp38, and two salt bridges (Glu74-Arg97 and Glu77-Lys 94) stabilizing the C-terminus of the molecule (Figure 1A and Supplementary Table S1). The Asp76-Lys41 salt bridge is affected by the D76N mutation related to hereditary systemic amyloidosis, resulting in the loss of the salt-bridge and leaving the positive charge of Lys41 uncompensated. In order to understand how this salt bridge contributes to the protein stability and amyloidogenicity, we prepared four β2m mutants (D76N, D76A, K41S, and D38N). The K41S mutant addresses the other side of the disease-related Asp76-Lys41 ion-pair, and thus it is useful to clarify the role of different effects and interactions in amyloidogenicity. D38N mutation on the other stabilizing charge-cluster is analogue with the D76N mutation, and we can verify how specific or general our findings are concerning the pathogenic mutation. Successful expression and renaturation to the correct fold of the recombinant β2m variants was achieved as confirmed by circular dichroism (CD) spectroscopy (Figure 1B). The spectra of the mutants were proven to be nearly identical with those of the WT β2m. In accordance with this, secondary structure analysis with the BeStSel tool [44,45] (https://bestsel.elte.hu, accessed on 23 October 2021). showed that native monomeric WT and mutant β2m proteins exhibited similar secondary structure composition, which is remarkably different from that of the acid-disordered WT protein and amyloid fibrils (Supplementary Table S2). Thermal stability and stability of β2m variants against chemical denaturant GdnHCl were tested by differential scanning calorimetry (DSC) and CD spectroscopy, respectively (Figure 1C,D). To estimate the melting points (*T*_m_) and unfolding enthalpy changes (Δ*H*) from DSC data, we used a two-state unfolding model, which fits well to the denaturation curves of β2m. The thermal unfolding of native β2m variants was reversible (at least 80% of the peak area with similar *T*_m_ was observed upon a second thermal scan). As shown in Table 1, *T*_m_ of D76N β2m is decreased by ~10 °C compared to WT, in agreement with previous reports [20,22,24]. In the case of K41S and D76A, *T*_m_ values are ~10 °C and ~7 °C lower, respectively, while the D38N mutant shows a moderate ~4 °C decrease in the melting point, similar to the finding of de Rosa et al. [24]. Using the van’t Hoff enthalpies of unfolding calculated from the DSC melting profiles, and the value 5.6 kJ/mol/K determined earlier for Δ*C*_p_ of unfolding of WT β2m [46], we estimated the thermal stability (Δ*G*_N-D_) of the β2m variants at a physiological temperature of 37 °C following the Gibbs-Helmholtz equation (Table 1). Δ*C*_p_ is mostly originated from the hydrophobic interactions [47], and because of the same or very similar 3D structure of the variants, it is reasonable to use the value of WT β2m for all variants. The results reveal that the Lys41-Asp76 ion-pair exhibits a significant stabilizing effect on β2m. The loss of stability is similar from both sides, i.e., by removal of the negative charge of Asp76 or the positive charge of Lys41. This proves that, indeed, the salt-bridge between these two side-chains itself plays a significant role in the stability of the molecule. In addition to Lys41, Valleix and colleagues [20] indicate Lys75 as another interacting positive charge for Asp76. Although we did not prepare a mutant to directly test the effect of Lys75, the symmetrical stability decrease for D76N and K41S mutants reveals that the real partner of Asp76 is solely Lys41 in the wild-type protein.

By removing most of the Asp76 side chain, the D76A mutant showed similar destabilization, however, with 3 °C higher *T*_m_ value compared to D76N. The removal of the negative charge of Asp38, disrupting another charge cluster of the molecule, resulted in moderately decreased stability (Table 1), revealing that Asp38 and its cluster have lower contribution to the conformational stability of β2m.

We investigated the stability of native β2m variants against GdnHCl by CD spectroscopy at a physiological temperature (37 °C). The unfolding of the structure was monitored by intensity changes of the CD signal at 212 nm, as a function of GdnHCl concentration (Figure 1D). While the Gibbs free energy change (Δ*G*_N-D_) of unfolding of D38N was slightly lower than that of WT β2m (~17 vs. ~19 kJ/mol), D76N, D76A, and K41S β2m variants were significantly less stable, showing Δ*G*_N-D_ values of ~12 kJ/mol at 37 °C (Table 1). This difference is also traceable in the midpoint concentration of GdnHCl in the unfolding experiments. These results are in accordance with earlier comparisons for WT and D76N [20,22]; however, they investigated the stability at lower temperature, 30 °C. 

Although GdnHCl stability measurements provided somewhat lower Δ*G*_N-D_ values than thermal denaturation, the same tendencies were observed for the variants. The 10–20% difference between the two methods suggests that both techniques are usable and address the thermodynamic stability of β2m.

### 3.2. The Effect of SDS and LPA on the Structure of β2m Monomers

**Figure 2:**
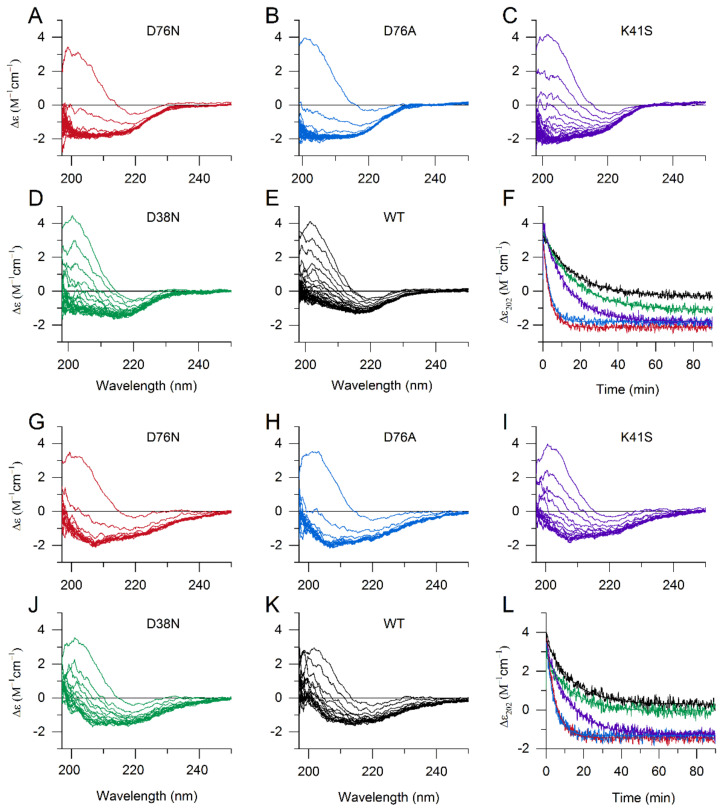
The effect of SDS and LPA on the structure of monomeric β2m variants. CD spectra of β2m variants were recorded in the presence of 500 μM SDS (**A**–**F**) and 300 μM LPA (**G**–**L**) in 50 mM Na-phosphate, 100 mM NaCl, pH 7.4. Spectra series of D76N (**A**,**G**), D76A (**B**,**H**), K41S (**C**,**I**), D38N (**D**,**J**), and WT (**E**,**K**) were recorded with 6 min steps. The kinetics of unfolding was also followed by Δε at 202 nm (**F**,**L**) in 500 μM SDS and 300 μM LPA, respectively.

Ookoshi et al. showed the amyloid-fibril-inducing effect of lysophosphatidic acid (LPA), which is a common molecule in biological fluids including blood, and its level is elevated in dialysis patients [35]. In our previous study, we found that it can destabilize the WT β2m monomer, induce fibril growth in a nucleation-dependent manner, and stabilize amyloid fibrils [36]. LPA is an amphiphilic molecule with a negatively charged headgroup, structurally similar to sodium dodecyl sulfate (SDS), which is also a well-known amyloid inducing molecule [48]. To study the effect of SDS and LPA on the native structure of β2m variants, we used CD spectroscopy. A total of 500 µM SDS at pH 7.4 exhibited an effect on the secondary structure of all variants, but D76N and D76A mutants seemed to be more sensitive than WT and the other mutants (Figure 2A–F). Similar effects were observed at 300 µM LPA (Figure 2G–L). D76N and D76A mutants visibly started to denature during the first CD spectrum scanning. An unfolding equilibrium was reached for these two mutants in 20 min in the presence of both 500 μM SDS and 300 μM LPA (Figure 2F,L). In the presence of 250 µM SDS, D76N and D76A showed moderate changes, while WT and D38N β2m exhibited only subtle spectral (and structural) changes (Supplementary Figure S1). We have to note that the CD spectrum reflects an average spectrum of all the conformers in the solution. Minor changes in the spectrum might come from unfolding of a smaller portion of the molecules. Regarding the kinetics of denaturation, K41S showed a significantly slower unfolding compared to D76N and D76A variants (Figure 2F,L; Supplementary Table S3). While the same ion-pair is disrupted in all three mutants, the remaining charge is positive in D76N and D76A and negative in K41S, which can explain the different interactions with the negatively charged headgroups of LPA and SDS. This reveals that the single uncompensated positive charge appearing at Lys41 in the D76N molecule might drastically change (enhance) the interaction of native β2m with negatively charged molecules, phosphoglycans, lipids, and membranes, which previously have been shown to affect WT β2m [32,33,34,35,36,49,50]. Intriguingly, the charge difference at position 76 mainly affects the kinetics of denaturation, because the final spectrum of K41S in the equilibrium is similar to D76N, D76A (Figure 2). This suggests that the charge difference might have a different effect on the partially unfolded state. We analyzed the CD spectra of the variants for the secondary structure contents after reaching the equilibrium denatured state in the presence of SDS and LPA using the BeStSel webserver [44,45] (Supplementary Table S4). The results revealed that β2m variants are partially unfolded with significant β-sheet content. For all the variants, both in the presence of 500 μM SDS and 300 μM LPA, we can see some α-helix content. It is known that SDS induces helix formation at moderate concentrations [51], and the LPA molecule has similar amphiphilic properties. β-sheet content is 15–20% lower in the partially unfolded state compared to native WT (Supplementary Table S4 vs. Table S2) in the presence of 500 μM SDS, depending on the variant (Supplementary Table S4). WT and D38N were less disordered than the other mutants. In the presence of 300 μM LPA, a similar decrease was observed in the β-sheet content; however, the spectral shape was clearly different from that measured in SDS, suggesting some structural differences. It is not clear how amyloidogenic the SDS- and LPA-denatured states are, but both the nucleation and elongation of amyloid fibrils are preferred under these conditions (see below).

### 3.3. Interactions of β2m Variants with Extracellular Matrix Proteins

**Figure 3:**
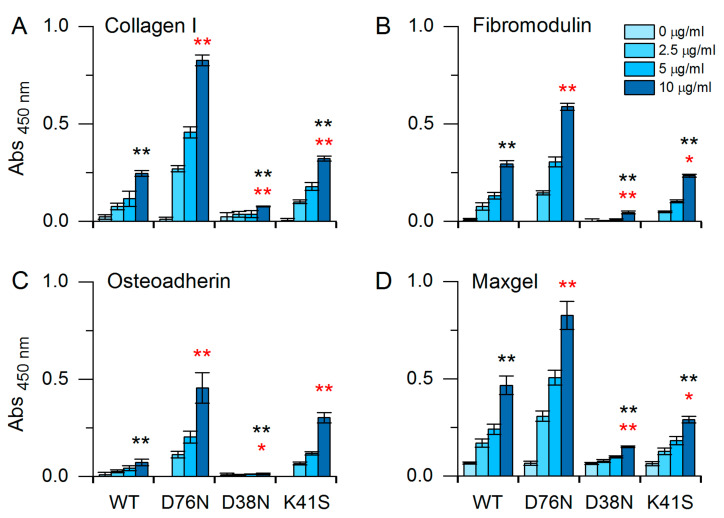
Interaction of β2m variants with ECM proteins, as collagen I (**A**), fibromodulin (**B**), osteoadherin (**C**), and maxgel (**D**) were measured by ELISA. Maxgel includes collagens, laminin, fibronectin, tenascin, elastin, and a number of proteoglycans and glycosaminoglycans. A dilution series (0, 2.5, 5, and 10 µg/mL) of β2m proteins was added to the microplate, which was previously coated with ECM proteins (10 µg/mL). β2m binding was detected using anti-human β2m and HRP conjugated goat anti-mouse secondary antibody. Data are mean absorbance values ± S.E.M., derived from three independent experiments. * *p* < 0.05, ** *p* < 0.01, two sample *t*-test, black asterisks: D76N vs. all, red asterisks: WT vs. all.

To investigate the different interaction network of the WT, D76N, D38N, and K41S β2m, ELISA experiments were carried out with extracellular matrix (ECM) proteins, such as collagen I, fibromodulin, osteoadherin, and Maxgel, which is a mixture of ECM proteins, proteoglycans, and glycosaminoglycans. ECM proteins were coated in microtiter plates for 1 h in 10 µg/mL concentration. The β2m mutants were added in dilution series (0–2.5–5–10 µg/mL). β2m variants showed significant binding affinity to ECM proteins in a concentration-dependent manner (Figure 3). Because the primary antibody exhibited the same affinity to all β2m variants, the observed differences must have come from different affinities of β2m variants to ECM proteins. Among them, D76N β2m showed the highest affinity, significantly higher than that of any other variants (Figure 3). The affinity of the WT protein was more dependent on the actual ECM protein, showing the least affinity for osteoadherin, while D38N generally showed the lowest affinity for any of the ECM proteins. Despite the low affinity of WT β2m, collagen has been found to play an essential role in the amyloid deposition of WT β2m in the bones and ligaments in the case of DRA [52]. The explanation lies in the highly elevated concentration of β2m in DRA, shifting the equilibrium towards binding. Moreover, ΔN6 N-terminal truncated β2m variant showed a tenfold higher affinity for collagen and might be a promoter of amyloidogenesis of WT β2m [53]. In the hereditary systemic amyloidosis, D76N β2m shows normal serum concentrations and exhibits visceral deposition [20]. Seemingly in contrast with this, we observed that D76N β2m has a higher affinity for collagen I than WT β2m has. However, the lower concentration of D76N in the patients and our finding that D76N generally has higher affinity to various ECMs can explain the different localization of amyloid deposits in the two diseases.

### 3.4. Amyloid Fibril Formation of β2m Variants Studied by ThT Fluorescence

#### 3.4.1. Amyloid Fibril Growth Induced by SDS and LPA in the Presence of Seeds

**Figure 4:**
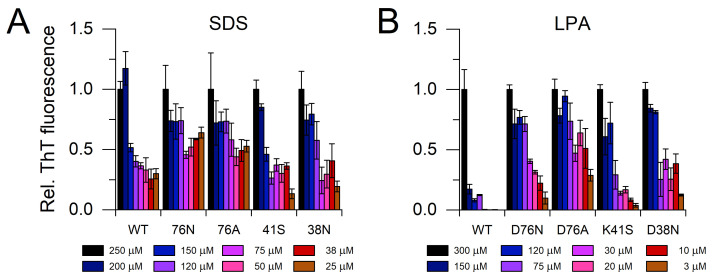
Amyloid fibril assembly of mutants and WT β2m, followed by ThT fluorescence. The measurements were carried out in the presence of 25–250 μM SDS (**A**) and 3–300 μM LPA (**B**) at 0.1 mg/mL protein concentration in a 50 mM Na-phosphate, 100 mM NaCl, 10 µM ThT, pH 7.4 buffer at 37 °C. The reactions were induced by the addition of 5 μg/mL seeds. ThT fluorescence intensities were normalized to the value at maximal SDS or LPA concentration of the corresponding β2m variant. Mean ± S.E.M. are shown.

We studied the amyloid fibril formation of the β2m variants at pH 7.4 in the presence of various concentration of SDS and LPA. It was shown earlier that SDS and LPA induce fibril growth of WT β2m and stabilize amyloid fibrils [35,36,40,48]. The polymerization was analyzed by thioflavin T (ThT) fluorescence after one week incubation at 37 °C (Figure 4). The protein concentration was 0.1 mg/mL, and the reaction was induced by 5 μg/mL seeds. The experiments were carried out in the presence 25–250 μM SDS and 3–300 μM LPA at 37 °C. At 250 μM SDS and 300 μM LPA, all variants polymerized fully, resulting in high ThT fluorescence intensities. Both SDS and LPA were proven to be the most effective for D76N and D76A β2m in inducing elongation of amyloid fibrils. There was a difference between the effect of SDS and LPA; the latter seemed to be less effective, especially on WT and K41S β2m.

#### 3.4.2. The Effect of Polyphosphate and SDS on Nucleation of Amyloid Formation

**Figure 5:**
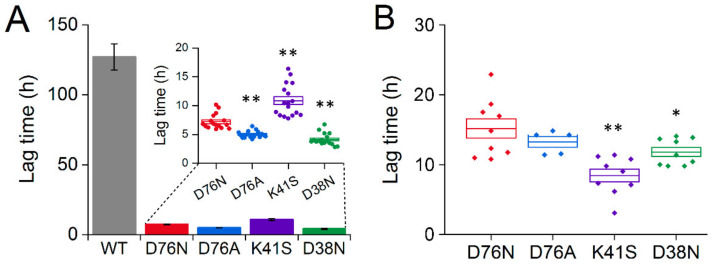
Average lag time of the amyloid assembly of β2m mutants and WT, with no addition of seeds, in the presence of poly-P and SDS. (**A**) The fibrillization of 0.3 mg/mL β2m solution was induced by 0.1 mM poly-P under continuous agitation. For a better comparison, the inset panel shows the lag time of β2m mutants, which occurs in a significantly shorter time scale than in the case of WT. (**B**) Lag times of spontaneous fibril formation of β2m mutants in the presence of 500 μM SDS. For all mutants, individual experimental points, mean, ± S.E.M. are presented, * *p* < 0.05, ** *p* < 0.01, two-sample *t*-test, compared to D76N. We have to note that in (**A**), D38N, and in (**B**), K41S were significantly lower than all the other variants, (*p* < 0.01, not shown in the figure).

It has been shown previously that poly-P induces amyloid formation of WT β2m [54]. We investigated the effect of poly-P on the β2m variants at a protein concentration of 0.3 mg/mL with no addition of seeds under continuous agitation. The reactions were followed by ThT fluorescence in a plate reader for 24 h or longer. All mutants showed a significantly shorter lag time for fibril formation in the presence of 0.1 mM poly-P than the WT β2m (Figure 5A and Supplementary Figure S2). Regarding the mutants, the results correlated with the charge at the site of mutation, i.e., positive charges of the related ion-pairs remained uncompensated by the D76N, D76A, or D38N mutations, possibly attracted by the negatively charged poly-P more than the negative charge of Asp76 side chain in the K41S variant (Figure 5A). An interesting result is that the D38N variant was even more sensitive to poly-P than D76N and D76A, which suggests that the effect might not be specific for the position of the mutation. The less negative overall net charge of the D76N, D76A, and D38N molecules compared to the WT protein might explain why poly-P is more effective on these; however, K41S, being the most negatively charged variant, still exhibits much higher amyloid forming tendency than the WT protein. This finding can be explained with the highly decreased stability of native K41S compared to WT β2m. These results are in agreement with and further support the previous finding that, at neutral pH, poly-P makes non-specific interactions with WT β2m through the strong Hofmeister effects of the condensed phosphate groups. This interaction can affect the hydration and stability of the interplaying native, disordered, and amyloid states [54].

We also studied the amyloid formation of the β2m variants in the presence of 500 μM SDS, with no addition of any preformed seeds. All variants of β2m, except WT, formed amyloid fibrils with lag times shorter than 24 h (Figure 5B and Supplementary Figure S3). Surprisingly, the shortest lag times were observed for K41S β2m, while the D38N mutant was closer to the D76N and D76A variants. This result is different from that observed in the presence of poly-P, which might be explained by the difference between the properties of the two additives. The poly-P used in our experiments has a negatively charged long chain consisting of 60–70 phosphate groups, while dodecyl-sulfate is an amphiphilic molecule having a single strong negatively charged headgroup with a hydrophobic 12-carbon tail, resulting in a different contribution of ionic and hydrophobic interactions.

### 3.5. Equilibrium Monomer Concentration of the Fibril Solutions

The structural stability of amyloid fibrils can be characterized by the concentration of the monomers in equilibrium with the fibrils. A low monomer concentration indicates an equilibrium shifted to the amyloid form, i.e., high stability of amyloid fibrils. In the case of fibrils with high stability, the remaining equilibrium monomer concentration might be very low, making it difficult to determine. Instead of this, denaturation with chemical denaturant (see Section 3.6) or thermal denaturation [40,55] are often used to measure the stability in the transition region, and extrapolations are made for the standard free-energy of association at the zero denaturant concentration or to physiological temperature, respectively. However, we made an attempt to characterize fibril stability by direct measurement of monomer concentration. 

To assess the effect of additives on the polymerization of β2m variants, we compared the equilibrium monomer concentration of β2m samples aggregated in the presence of different concentrations of LPA and SDS at neutral pH at 37 °C. The reactions were induced by the addition of seeds. Using ultracentrifugation (~105,000× *g* for 60 min), it was possible to spin down all the aggregated material and have the monomeric protein in the supernatant. Earlier, we used a quantitative ELISA kit to determine the monomer concentration [46,56], which might be affected by the presence of the additives used in the present study. We quantified the monomer concentration by the intrinsic, Trp fluorescence of β2m. Trp residues were selectively excited at 295 nm, and the emission spectra were recorded in the range of 310–400 nm. Area under the curve was calculated for each spectrum. Calibration curves were prepared for standard β2m monomeric solutions (0.20–100 μg/mL) using the same buffer conditions as in the fibrillization experiments. Calibration curves were fairly linear with the protein concentration. The concentration limit of the method was ~0.1 μg/mL concentration (~0.01 μM). 

**Figure 6:**
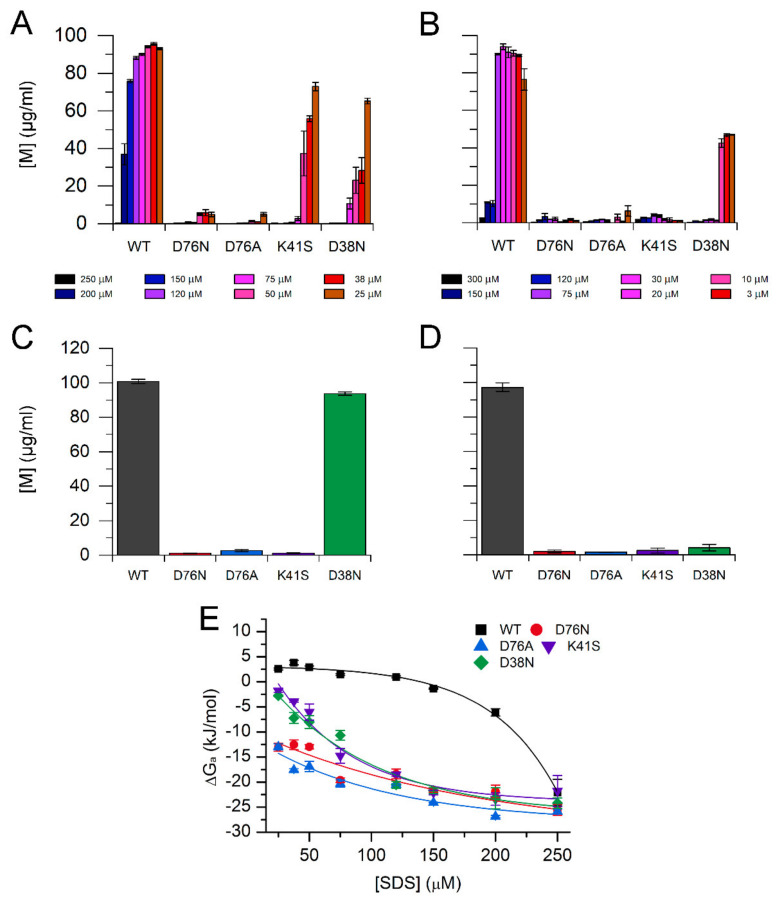
Equilibrium monomer concentrations in WT and mutant β2m fibril solutions. Amyloid fibrils were grown in the presence of 25–250 μM SDS (**A**) and 3–300 μM LPA (**B**) in a buffer of 50 mM Na-phosphate, 100 mM NaCl, pH 7.4, by the addition of 5 μg/mL fibril seeds. Spontaneous amyloid formation of the variants was studied in the lack of seeds and any additives after one week incubation at 37 °C with continuous agitation (**C**). In a similar experiment, monomer concentrations were tested in the presence of 30 μM LPA after one week incubation (**D**). In all experiments, an overall protein concentration of 0.1 mg/mL was used. Monomer concentrations were determined by Trp fluorescence after ultracentrifugation of aggregated material (Supplementary Figures S4 and S5, and Materials and Methods). (**E**) Gibbs free energy of fibril association calculated from the equilibrium monomer concentrations as a function of SDS concentration. Mean ± S.E.M. are presented.

As shown in Figure 6, the WT protein displays a significantly different profile compared to the mutant forms, containing higher levels of monomer protein in the presence of lower concentrations of additives. In contrast to the WT form, D76 mutant samples were found to barely contain any soluble monomer protein, even at the lowest concentrations of LPA and SDS. When K41S β2m aggregated in the presence of LPA (Figure 6B and Supplementary Figure S4F–J), the polymerization of the protein showed similar characteristics to D76N and D76A mutants: the monomeric protein solution underwent an almost 100% polymerization during the incubation time, even at the lowest additive concentrations. By contrast, when the aggregation of K41S β2m was induced in an SDS-containing buffer (Figure 6A), this variant displayed a sensitivity profile distinct from the one observed in WT and D76 samples, but similar to the D38N mutant. β2m D38N showed intermediate sensitivity in the presence of both LPA and SDS. In addition to the molecular differences between SDS and LPA, their different effect can be explained by the fact that LPA forms micelles at the concentrations studied, while SDS mostly acts as monomer, below its critical monomer concentration (CMC) [36,48].

It has been reported that D76N β2m is able to form amyloid fibrils even without the addition of preformed amyloid fibrils or any additives to the protein solution at physiological pH, while WT β2m does not polymerize under such conditions even at high protein concentrations with long incubation time [20,53]. Therefore, we tested how the monomer concentration of β2m samples change after 1 week of incubation without the addition of preformed amyloid seeds and detergents under strong agitation. We found that the mutations affecting the K41-D76 salt bridge increased the aggregation propensity of the β2m molecule, resulting in low remaining monomer concentrations (Figure 6C, Supplementary Figure S5A), whereas D38N mutation did not cause any significant decrease in the monomer concentration, similar to the WT protein. However, in a similar experiment, the presence of 30 μM LPA increased the aggregation propensity of D38N to the level of the other mutant forms (Figure 6D, Supplementary Figure S5B). This result is similar to the one we could observe in the seeded reactions at such LPA concentrations (Figure 6B).

Conformational stability of the amyloid fibrils, i.e., the standard Gibbs free energy of association (Δ*G*_a_), was calculated from the equilibrium monomer concentrations as described in Section 2.7.1. We applied the linear polymerization model [39,40,41,42], assuming that the equilibrium constant for adding a monomer to the end of a chain, *K*_a_, is independent of length. Figure 6E shows the stability of fibrils of β2m variants as a function of SDS concentration. D76A and D76N were proven to have the highest stability at any SDS concentration. At 250 μM SDS, all the variants formed stable fibrils. With decreasing SDS concentration, WT β2m fibrils steeply lost their stability and hardly formed. D38N and K41S fibrils exhibited decreased stability below 100 μM SDS, losing their stability at 25 μM. D76N and D76A fibrils showed decreased but still significant stability even at 25 μM SDS (Figure 6E). The equilibrium monomer concentrations in the presence of LPA were more scattered; we only gave a qualitative description above and did not calculate Δ*G*_a_ values.

We also calculated the stability from the remaining monomer concentration for D76N and D76A β2m fibrils in the absence of any additives and seeds after one week incubation under physiological conditions with strong agitation at 37 °C. Δ*G*_a_ was found to be 17.5 ± 0.4 and 14.9 ± 0.7 kJ/mol for D76N and D76A fibrils, respectively. These values are lower than that was reported by Natalello et al. for D76N fibrils [27]; however, they measured the stability at much higher protein concentration (100 μM vs. 8.4 μM) in the presence of preformed seeds by GdnHCl denaturation at room temperature.

### 3.6. Stability of β2m Amyloid Fibrils Investigated by GdnHCl Denaturation

**Figure 7:**
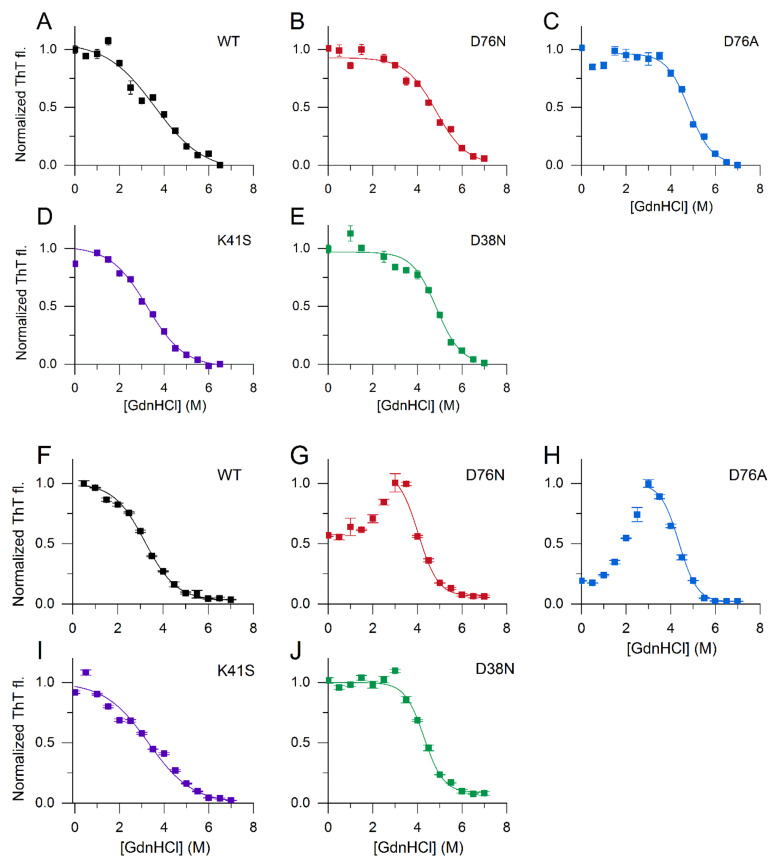
Stability of β2m amyloid fibrils against GdnHCl, monitored by ThT fluorescence. Amyloid fibrils were grown, and their stability was tested in the presence of 250 μM SDS (**A**–**E**) and 300 μM LPA (**F**–**J**) at 37 °C. Amyloid fibrils were incubated at various concentrations of GdnHCl for 24 h, and the remaining ThT fluorescence was measured as described in Section 2.7.2. Data were normalized for the maximal ThT intensities. The sigmoidal curves are guides to the eye.

To investigate the thermodynamic stability of β2m amyloid fibrils, the remaining ThT fluorescence was measured as a function of the concentration of the chemical denaturant, guanidium hydrochloride (GdnHCl). After overnight growing in seeded reactions in the presence of 250 μM SDS or 300 μM LPA at neutral pH at 37 °C, the amyloid fibrils were incubated in GdnHCl solutions of various concentrations for 24 h. Figure 7A–E shows the difference between the stability of the WT, D76N, D76A, K41S, and D38N β2m fibrils grown in the presence of 250 μM SDS. Thermodynamic stability was calculated using the linear polymerization model [39,40,41,42]. The WT and K41S fibrils showed lower stability; their estimated Δ*G*_a_^0^ values were –17.3 ± 0.5 and –15.1 ± 0.6 kJ/mol, respectively, and the observed midpoint concentrations were lower than that of the mutants where Asp residue was replaced (Table 2). D76N and D76A exhibited –22.1 ± 0.3 and –24.2 ± 1.8 kJ/mol stabilities. D38N β2m was somewhat less stable, with –19.2 ± 3.0 kJ/mol free-energies. 

Similar effects could be observed in the case of amyloid fibrils grown in the presence of 300 μM LPA (Figure 7F–J). The conformational stability of D76N, D76A, and D38N mutants proved to be significantly higher than that of WT and K41S β2m. D76N and D76A showed an interesting phenomenon: their ThT fluorescence increased even with increasing GdnHCl at moderate concentrations, before fibrils started to dissociate above 3 M GdnHCl. The fibrils were grown up undoubtedly with low remaining monomer concentration. The effect might come from a re-arrangement of fibril filaments, increasing the surface for ThT binding, or an unknown reason. These stability values were about half of those reported for D76N and WT β2m in the presence of 20% TFE against GdnHCl denaturation at room temperature [27] and WT β2m fibril stability measured at an acidic pH of 2.5 [41]. 

### 3.7. Synergy of Diverse Effects Is behind the Amyloidogenicity of D76N β2m, Causing Hereditary Systemic Amyloidosis

Recently, several studies have attempted to explain the remarkable differences found between the pathology of hereditary systemic β2m amyloidosis and dialysis-related amyloidosis. A number of studies investigated the properties of the unaccompanied D76N β2m molecule and compared them to WT β2m and carefully designed mutants. Native structures were found to be similar by X-ray crystallography and NMR [20,22,24,53]. Besides its significantly decreased stability, the amyloidogenic propensity of native D76N β2m was attributed to alterations in conformational dynamics and unfolding-refolding kinetics [22,23,24,28,57,58,59]. By finding that it is not the overall net charge or pI of the protein, nor the overall charge distribution on the β2m surface, De Rosa et al. speculated that “the decreased stability and remarkable aggregation trends of the D76N β2m mutant must be the result of specific yet uncharacterized properties, strictly linked to the structural location of the protein 76 site” [24]. Natalello et al. [27] determined the stability of D76N amyloid fibrils and compared it to WT β2m fibrils in 20% TFE against GdnHCl. They found that D76N fibrils were significantly more stable than WT ones. 

Because at neutral pH, WT β2m does not form amyloid fibrils, even at elevated concentrations, without additives, specific interactions with extracellular matrix molecules, such as type I collagen, glycosaminoglycans (GAGs), and proteoglycans (PGs) are associated with β2m amyloid deposition [32,33,52,60,61]. The possible role of lysophospholipids (LPLs) and non-esterified fatty acids (NEFAs) was also pointed out [35,36,62]. Although D76N β2m was proven to form amyloid fibrils at neutral pH without any inducing agents under sheer forces [22], we believe that the molecular environment in the patients, similarly to the case of WT β2m in DRA, may strongly affect its amyloid formation. 

Using various β2m mutants, we studied the contribution of diverse effects on the aggregation of D76N β2m, such as net charge, conformational stability of the native and amyloid state, effect of additives, and binding to interaction partners. Out of the numerous charged residues of β2m, only a few of them formed intramolecular ion-pairs (Supplementary Table S1). The Asp76-Lys41 ion-pair and the cluster centered on Asp38 are formed by sequentially distant side-chains and therefore expected to have significant contribution to the overall stability and fold of WT β2m. Comparison of the D76N, D76A, and K41S mutants revealed similar, highly decreased stability, suggesting that the decreased conformational stability of D76N β2m exclusively comes from the loss of the Asp76–Lys41 ion-pair (Figure 1 and Table 1). 

The remaining uncompensated positive charge of Lys41 in D76N enhanced the interactions with the negatively charged amphiphilic SDS and LPA molecules, inducing a rapid partial unfolding. The K41S mutant, having similarly decreased conformational stability, showed a much slower yet similar level of unfolding (Figure 2, Supplementary Tables S3 and S4). The SDS- and LPA-induced partially unfolded states are predisposed to form amyloid fibrils. D76N and D76A formed more stable fibrils than K41S, D38N, or WT β2m, especially at low SDS and LPA concentrations in seeded reactions (Figure 4, Figure 5 and Figure 6, Supplementary Figures S3 and S4). Intriguingly, in the presence of 500 μM SDS, without seeds, the lag-time of amyloid nucleation was proven to be the shortest for K41S, while D38N was closer to D76N and D76A. WT β2m did not form fibrils during the experiment time (Figure 5B). This suggests that the amyloid nucleation process in the partially unfolded state induced by 500 μM SDS does not correlate with the net charge (−3.2, −2.2, and −1.2 charges for K41S, WT, and D76N/D76A/D38N mutants, respectively), and the site of residue 76 is not distinguished. In contrast to these, lag-times of poly-P induced amyloid formation were shorter for the Asp mutants D76N, D76A, and D38N than for K41S (Figure 5A). However, all the mutants exhibited much shorter lag-times than WT β2m, which might come from the higher conformational stability of the native state. Again, the net charge is not crucial in this process because the K41S variant still readily forms amyloid fibrils in the presence of poly-P at pH 7.4. Moreover, despite its unfavorable net charge, K41S spontaneously aggregated under strong agitation in the lack of additives at neutral pH (Figure 6C). Under slightly destabilizing conditions (30 μM LPA), D38N β2m also aggregated without seeds (Figure 6D). 

Native D38N β2m has only slightly decreased stability compared to WT β2m. This indicates that the destabilization of the native state is a rather crucial factor in the aggregation propensity, and once partially unfolded conformers are populated, the difference in a few charges is less important in the nucleation and elongation of fibrils (albeit the thermodynamic stability of the fibrils will be different). 

Regarding fibril stability, the cryo-EM structure of the WT β2m fibrils grown at pH 2.5 shows Asp76 in the L-shaped subunit core, in a region stabilized by hydrophobic interactions [29]. While the Asp side-chain is protonated and thus neutral at pH 2.5, it has a negative charge at pH 7.4, which is probably unfavorable in WT, K41S, and D38N β2m fibrils, destabilizing them (Table 2). However, even at pH 7.4, the neutral Asn76 side-chain of the pathogenic mutant is compatible with the structure observed at pH 2.5.

β2m variants showed significant binding affinity to extracellular matrix proteins (Figure 3). D76N β2m showed the highest affinity, significantly higher than that of any other variants. The affinity of the WT protein was more dependent on the actual ECM protein, showing a high affinity for “Maxgel”, which is a mixture of extracellular components and might be a good model for osteoarticular tissues involved in the depositions in DRA.

**Figure 8:**
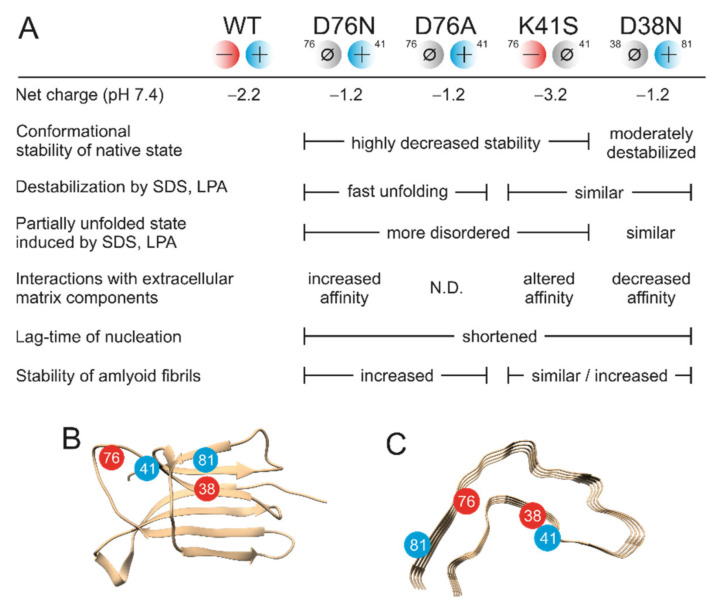
Diverse effects of point mutations on the native state and amyloid formation of β2m. (**A**) Comparison of various molecular properties of β2m mutants studied in this work to those of WT β2m. Net charge was estimated using the pKa values reported by Mangione et al. [22], based on calculations with the software BLUUES [63]. (**B**,**C**) Native and amyloid (pH 2.5) structure of WT β2m, respectively. Relative position of the charged side-chains related to this work are represented with red and blue circles.

Taken together, all the above observations show that the exceptional amyloidogenicity of D76N pathogenic β2m variant (relative to WT β2m) is realized by the synergy of diverse effects of destabilized native structure, higher sensitivity to negatively charged amphiphilic molecules and polyphosphate, more effective fibril nucleation, higher conformational stability of fibrils, and elevated affinity for extracellular components, including ECMs (Figure 8). 

## 4. Conclusions

In this work we investigated the effect of the pathogenic D76N mutation on the native and amyloid states of β2m, as well as the altered intermolecular interaction network of the mutant protein. To test how site-specific the observed effects are for the mutation and to understand the molecular background of the observed effects, we designed further mutants: D76A, K41S, and D38N. Using a variety of biophysical techniques, we found that the exceptional amyloidogenicity of D76N β2m is realized by the deleterious synergy of diverse effects of destabilized native structure, higher sensitivity to negatively charged amphiphilic molecules (e.g., lipids) and polyphosphate, more effective fibril nucleation, higher conformational stability of fibrils, and elevated affinity for extracellular components, including extracellular matrix proteins. These observations can provide an explanation for the different pathology of DRA and hereditary systemic β2m amyloidosis. Moreover, D76N β2m is an ideal model protein to study the effect of point mutations on the amyloidogenicity of proteins, revealing all the various molecular properties and target-points a mutation might affect, and should be taken into consideration, e.g., in the study of hereditary mutants of amyloidogenic proteins.

## Figures and Tables

**Table 1:** Stability of native β2m variants, as tested by DSC (thermal denaturation) and by chemical denaturant, GdnHCl.

Protein	*T*_m_ (°C) ^a^	Δ*H*_v_ (kJ/mol) ^b^	Thermal Denat. Δ*G*_N-D_ (kJ/mol) ^c^	GdnHCl Denat. Δ*G*_N-D_ (kJ/mol) ^d^
WT	65.5 (±0.02)	367.0 (±1.4)	24.0 (±0.5)	18.9 (±0.8)
D76N	55.9 (±0.03)	283.2 (±2.5)	13.2 (±1.6)	12.0 (±0.5)
D76A	58.3 (±0.04)	283.1 (±3.2)	14.3 (±0.2)	11.1 (±1.3)
K41S	55.8 (±0.07)	311.9 (±7.3)	14.8 (±0.5)	11.3 (±1.0)
D38N	61.3 (±0.09)	329.0 (±2.7)	18.8 (±0.3)	16.9 (±1.7)

^a^ Melting point (*T*_m_), ^b^ van’t Hoff enthalpy (Δ*H*_v_), and ^c^ Gibbs free energy change (ΔG_N-D_) of thermal unfolding calculated at 37 °C for β2m variants from DSC melting curves. ^d^ Stability of β2m variants against GdnHCl-induced denaturation at 37 °C.

**Table 2:** Stability of amyloid fibrils of β2m variants.^a^

	250 μM SDS		300 μM LPA	
	Δ*G*_a_^0^ (kJ/mol)	*C*_m_ (M)	Δ*G*_a_^0^ (kJ/mol)	*C*_m_ (M)
WT	–17.3 (±0.5)	3.5	–11.7 (±0.2)	3.5
D76N	–22.1 (±0.3)	4.8	–23.6 (±0.7)	4.8
D76A	–24.2 (±1.8)	4.7	–25.6(±3.2)	4.7
K41S	–15.1 (±0.6)	3.6	–12.5 (±0.3)	3.6
D38N	–19.2 (±3.0)	4.9	–24.8 (±2.0)	4.9

^a^ Standard Gibbs free energy in the absence of denaturant (Δ*G*_a_^0^) and midpoint GdnHCl concentration (*C*_m_) of fibril association investigated at 37 °C. Fibrils were grown in the presence of 250 μM SDS or 300 μM LPA in 50 mM Na-phosphate, 100 mM NaCl, pH 7.4.

## Data Availability

The data in this study are readily available upon reasonable request to the corresponding author.

## Supplementary Materials

The following are available online at https://www.mdpi.com/article/10.3390/biology10111197/s1, Table S1: Ionic Interactions in β2m. Table S2: BeStSel analysis of CD spectra of β2m variants. Table S3: Kinetics of SDS- and LPA-induced unfolding. Table S4: Secondary structure in the presence of SDS and LPA. Figure S1: Effect of 250 μM SDS on the structure of native β2m variants followed by CD spectroscopy. Figure S2: The amyloid polymerization of β2m variants induced by 0.1 mM poly-P. Figure S3: The amyloid fibril formation of β2m variants induced by SDS in the lack of seeds. Figure S4: Equilibrium monomer concentrations followed by intrinsic fluorescence. Figure S5: Equilibrium monomer concentrations in the lack of seeds and additives were tested by Trp fluorescence of supernatants after ultracentrifugation.
