# Supplementary material for: Pathogenic D76N Variant of β2-Microglobulin: Synergy of Diverse Effects in Both the Native and Amyloid States
Source: Biology (Basel). 2021 Nov 17;10(11):1197. doi: 10.3390/biology10111197 (PMC8614874; doi:10.3390/biology10111197)
Supplement: Supplementary file 1 [file biology-10-01197-s001.zip › biology-1454484-supplementary.pdf]

Supplementary Materials

Pathogenic D76N Variant of β2-Microglobulin: Synergy of Diverse Effects in both the Native and Amyloid States

Éva Bulyáki, Judit Kun, Tamás Molnár, Alexandra Papp, András Micsonai, Henrietta Vadászi, Borbála Márialigeti, Attila István Kovács, Gabriella Gellén, Keiichi Yamaguchi, Yuxi Lin, Masatomo So, Mihály Józsi, Gitta Schlosser, Young-Ho Lee, Károly Liliom, Yuji Goto, József Kardos*
*Correspondence: kardos@elte.hu

Supplementary Table S1. Ionic Interactions in β2m.

Position1	Position2
**E36**	**R81**
**D38**	**R45**
**D38**	**R81**
**K41**	**D76**
R45	E47
K41	E50
E50	H51
**E74**	**R97**
**D76**	**R97**
**E77**	**K94**

Ionic interactions in β2m within 6 Å (PDB ID: 2YXF) using PIC server [1]. Ion-pairs consisting of sequentially distant side-chains are highlighted in bold.

Supplementary Table S2. BeStSel analysis of CD spectra of β2m variants[a]

β2m	α-helix (%)	β-sheet (%)	Others (%)	NRMSD
WT	0.0	51.3[b]	48.7	0.03
D76N	0.0	51.2	48.8	0.03
D76A	0.0	50.0	50.0	0.03
K41S	0.0	51.5	48.6	0.03
D38N	0.0	50.9	49.0	0.03
WT denat.	5.1	21.5	73.4	0.02
WT amyloid	2.5	57.6[b]	39.9	0.01
X-ray (2yxf)	0	47.9	52.0	

[a] CD spectra of β2m variants were analyzed at the BeStSel webserver (https://bestsel.elte.hu) for secondary structure composition [2,3]. To show the sensitivity of the CD spectrum to the structure of β2m, we provide the CD spectra of acid-denatured and amyloid fibril WT β2m as comparison. [b]β-sheets in native β2m consists of strands in antiparallel orientation, while amyloid fibrils have parallel β-structure which is reflected in spectral shape. For reference, X-ray structure of native WT β2m is also shown (PDB: 2yxf). NRMSD: normalized RMSD of spectral fitting [4].

Supplementary Table S3. Kinetics of SDS- and LPA-induced unfolding

	V_0 (SDS)[a] mdeg/min	V_0 (LPA)[a] mdeg/min
WT	-0.21	-0.24
D76N	-1.50	-1.10
D76A	-1.41	-1.13
K41S	-0.44	-0.36
D38N	-0.26	-0.21

[a] V_0 values of exponential decay fitting for the unfolding kinetics measured by CD at 202 nm in 500 μM SDS and 300 μM LPA.

Supplementary Table S4. Secondary structure of β2m variants in the presence of SDS and LPA[a]

	α-helix (%)	β-sheet (%)	Others (%)	NRMSD
		500 μM SDS		
D76N	8.9	32.1	59.0	0.0143
D76A	4.4	32.7	62.9	0.0148
K41S	4.6	27.6	67.8	0.0183
D38N	2.7	37.6	59.7	0.0199
WT	2.7	35.8	61.4	0.0311
		300 μM LPA		
D76N	5.0	30.4	64.6	0.0179
D76A	5.5	25.5	69.0	0.0231
K41S	10.0	30.2	59.8	0.0184
D38N	4.3	33.8	61.9	0.0208
WT	4.7	28.9	66.5	0.0361
X-ray (2yxf)	0	47.9	52.0	

[a] CD spectra of β2m variants measured in the presence of 500 μM SDS and 300 μM LPA were analyzed at the BeStSel webserver (https://bestsel.elte.hu) for secondary structure composition [2,3]. For reference, X-ray structure of native WT β2m is also shown (PDB: 2yxf). NRMSD: normalized RMSD of spectral fitting [4].

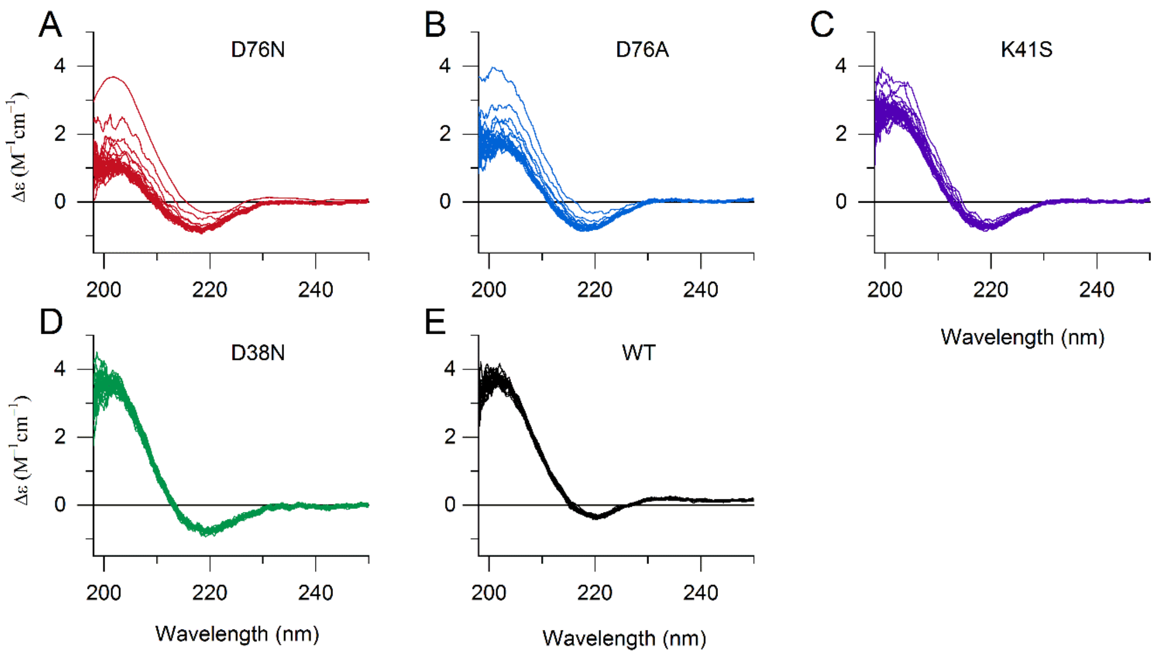

Supplementary Figure S1. Effect of 250 μM SDS on the structure of native β2m variants followed by CD spectroscopy. Spectra series were recorded with 6 min steps in the presence of 250 μM SDS in 50 mM Na-phosphate, 100 mM NaCl, pH 7.4.

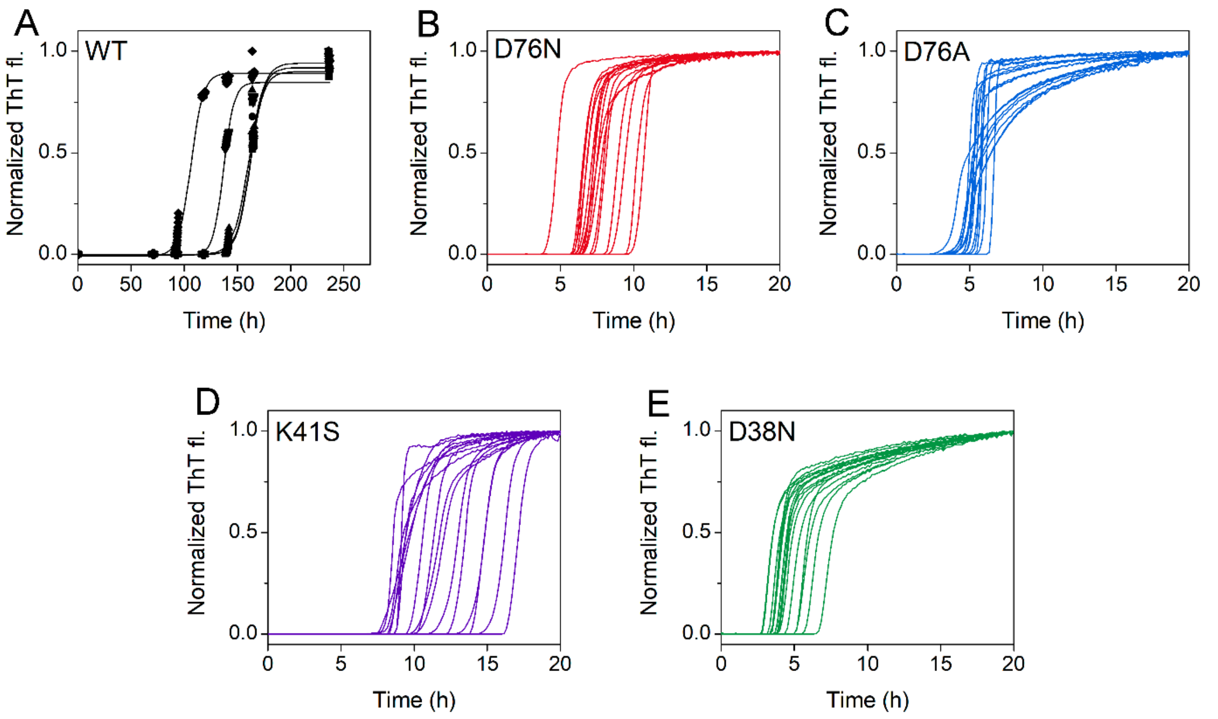

Supplementary Figure S2. The amyloid polymerization of β2m variants induced by 0.1 mM poly-P. A protein concentration of 0.3 mg/ml was used in 20 mM Tris, pH 7.4. Fibrillization was followed by ThT fluorescence and measured in spectrofluorometer (WT) or plate reader (mutants). ThT values were normalized to their maximum.

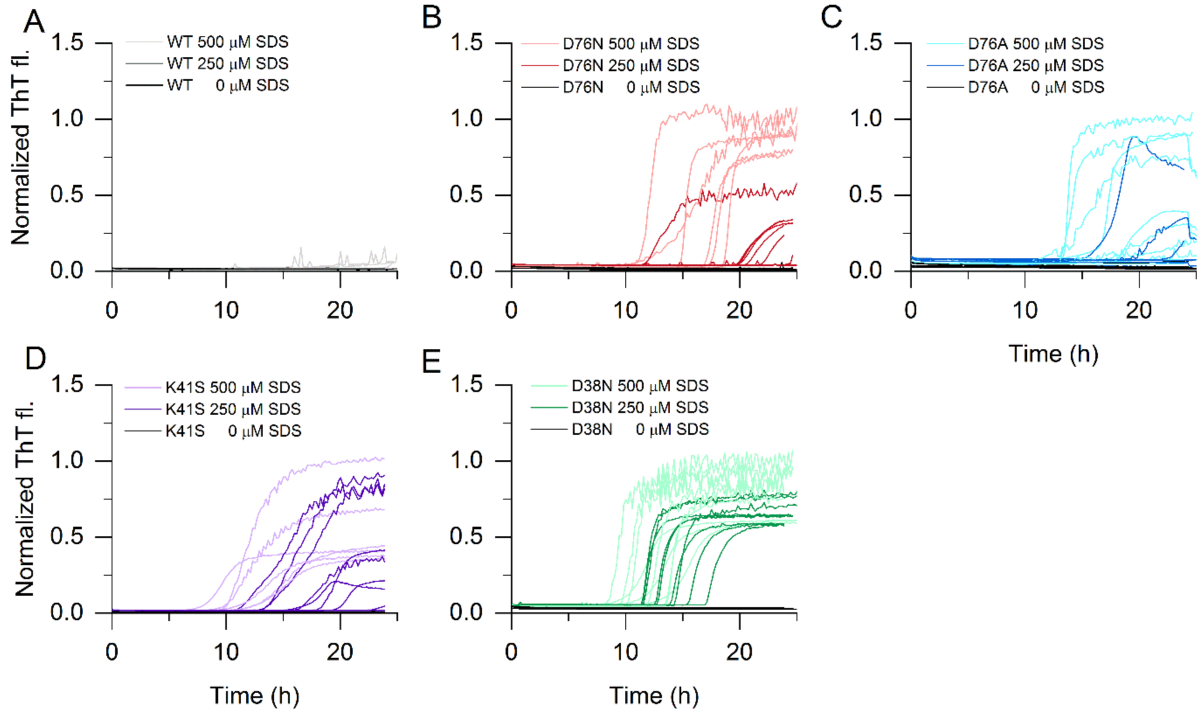

Supplementary Figure S3. The amyloid fibril formation of β2m variants induced by SDS in the lack of seeds. A protein concentration of 0.1 mg/ml was used in 50 mM Na-phosphate, 100 mM NaCl, pH 7.4. Fibrillization was followed by ThT fluorescence. Fluorescence intensities were normalized to the maximal value in the series.

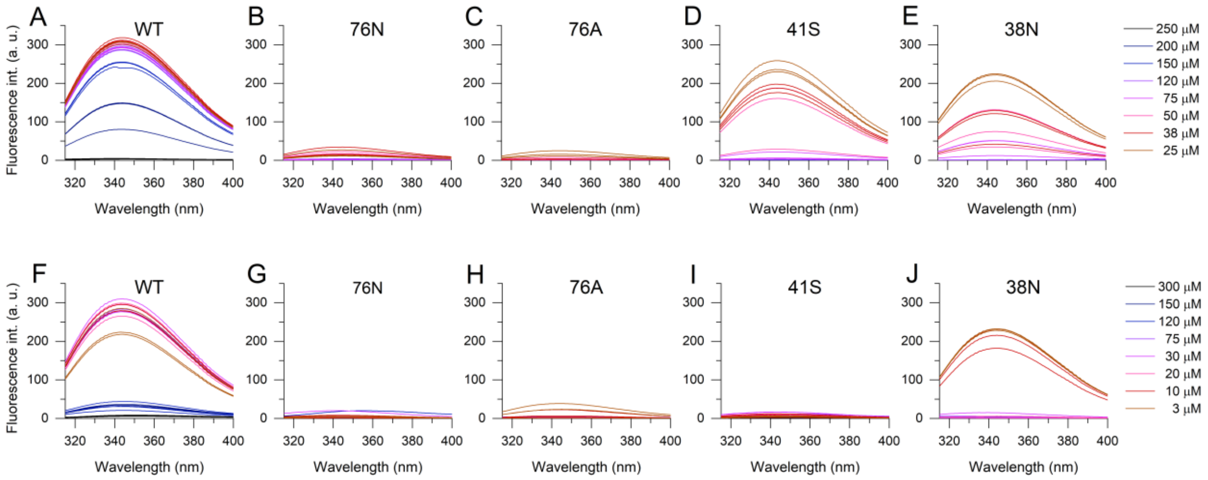

Supplementary Figure S4. Equilibrium monomer concentrations followed by intrinsic fluorescence. Trp fluorescence spectra of supernatants were recorded after ultracentrifugation of amyloid fibril solutions. Fibrils were grown at an overall protein concentration of 0.1 mg/ml in the presence of 25-250 µM SDS (A-E) and 3-300 µM LPA (F-J) for 48 hours by the addition of 5 µg/ml preformed fibril seeds in a 50 mM Na-phosphate, 100 mM NaCl, pH 7.4. Samples in triplicates are shown.

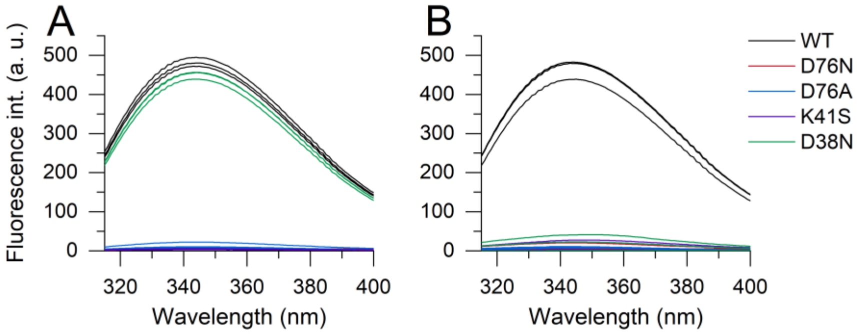

Supplementary Figure S5. Equilibrium monomer concentrations in the lack of seeds and additives were tested by Trp fluorescence of supernatants after ultracentrifugation. Spontaneous amyloid formation of the variants were studied in the lack of seeds and any additives after one week incubation at 37 °C with continuous agitation (A). In a similar experiment, monomer concentrations were tested in the presence of 30 μM LPA after one week incubation (B). In all experiments, an overall protein concentration of 0.1 mg/ml was used in 50 mM Na-phosphate, 100 mM NaCl, pH 7.4. Samples in triplicates are shown.

Supplementary references

1. Tina, K.G.; Bhadra, R.; Srinivasan, N. PIC: Protein Interactions Calculator. Nucleic Acids Res. 2007, 35, W473–W476, https://doi.org/10.1093/nar/gkm423.
2. Micsonai, A.; Bulyaki, E.; Kardos, J. BeStSel: From Secondary Structure Analysis to Protein Fold Prediction by Circular Di-chroism Spectroscopy. Methods Mol. Biol. 2021, 2199, 175–189, https://doi.org/10.1007/978-1-0716-0892-0_11.
3. Micsonai, A.; Wien, F.; Bulyaki, E.; Kun, J.; Moussong, E.; Lee, Y.H.; Goto, Y.; Refregiers, M.; Kardos, J. BeStSel: A web server for accurate protein secondary structure prediction and fold recognition from the circular dichroism spectra. Nucleic Acids Res. 2018, 46, W315–W322, https://doi.org/10.1093/nar/gky497.
4. Micsonai, A.; Wien, F.; Kernya, L.; Lee, Y.H.; Goto, Y.; Refregiers, M.; Kardos, J. Accurate secondary structure prediction and fold recognition for circular dichroism spectroscopy. Proc. Natl. Acad. Sci. USA 2015, 112, E3095–E3103, https://doi.org/10.1073/pnas.1500851112.
